# Editorial: Redox and Nitrosative Signaling in Cardiovascular System: From Physiological Response to Disease

**DOI:** 10.3389/fphys.2018.01538

**Published:** 2018-11-02

**Authors:** Mariarosaria Santillo, Pasquale Pagliaro

**Affiliations:** ^1^Dipartimento di Medicina Clinica e Chirurgia, Università di Napoli Federico II, Naples, Italy; ^2^Dipartimento di Scienze Cliniche e Biologiche, Università di Torino, Turin, Italy

**Keywords:** redox signaling, nitrosative signaling, cardiovascular system, oxidative stress, nitrosative stress, reactive oxygen species (ROS), reactive nitrogen species (RNS)

Reactive oxygen species (ROS) are highly reactive substances generated by the chemical utilization of oxygen inside the cells. High levels of ROS induce macromolecule damage leading to a variety of diseases, but controlled ROS generation play a role in redox-sensitive gene expression and cell signaling regulating physiological processes including cardiovascular functions.

The main scope of the present special issue was to reach a broad audience of scientists working in the field of cardiovascular redox biomedicine. We encouraged the submission of papers approaching the topic from different points of view and at different levels, from basic to translational research. Indeed, a collection of scientific reports and review articles with different approaches contributed to the special issue highlighting interesting aspects of redox biology in several cardiovascular fields. In addition, the research topic includes an intriguing hypothesis article by Davies reporting that adaptation of the cardiovascular system to exercise training is one of the most significant examples of adaptive homeostasis: defined as “*The transient expansion or contraction of the homeostatic range in response to exposure to sub-toxic, non-damaging, signaling molecules or events, or the removal or cessation of such molecules or events*.” Endurance training involves the generation of low levels of free radicals and hydrogen peroxide which do not cause damage, but rather activate signal transduction pathways, such as Nrf2 and NFκB, to induce mitochondrial biogenesis—the foundation of increased exercise endurance. As with other examples of adaptive homeostasis, the effects are transient, lasting only as long as the training is maintained. Unfortunately, the ability to adapt to exercise training declines with age, perhaps as a result of impaired Nrf2 and NFκB signaling, as does adaptive homeostasis capacity in general.

Several original articles and reviews included in the special issue emphasize the role of mitochondria in cardiac activity both in physiologic and pathological conditions. The mini-review by Pagliaro et al. deals with the role of mitochondria in ischemic and pharmacological cardiac postconditioning. The main interesting aspect of this work is the deepening of the signaling pathways converging on mitochondria able to preserve many of the mitochondrial functions after ischemia/reperfusion. In particular, the role of mitochondrial components like connexin 43, mitochondrial K_ATP_ channels and mitochondrial permeability transition pore in cardioprotective effects of postconditioning are widely highlighted. Another review article by Penna et al. examines the role of chaperones in the heart and the redox aspects that can influence cardiac chaperone function, especially within mitochondria. Chaperones are stress proteins involved in the adaptive response to stress conditions and in this review are discussed the redox-dependent regulation of chaperones underlying the cardiac ischemia/reperfusion injury as well as cardioprotection.

The original article by Boengler et al. point outs that depending on their amounts, reactive oxygen species (ROS) may either be detrimental [in myocardial ischemia/reperfusion (IR) injury] or protective (ischemic preconditioning, IPC). Here, the authors addressed the role of the ROS-producing enzyme p66shc in IR and IPC. Following IR (not IPC), p66shc translocated into cardiac subsarcolemmal mitochondria and this was associated with increased ROS formation. However, p66shc-deficient hearts showed similar infarct sizes after IR and effective cardioprotection by IPC suggesting that p66shc-derived ROS do not contribute to IR injury per se and are not involved in the cardioprotection by IPC. Schiattarella et al. showed that animals with mitochondrial A-kinase anchor protein (AKAP1) knockdown or knockout are more sensitive to TAC, an experimental model to induce pressure overload, heart hypertrophy, cardiomyocyte apoptosis and heart failure. Indeed, animals Akap 1^−/−^ showed increased levels of ROS, apoptotic markers such as short caspase-3, and TUNEL positive cells in cardiac tissues. In particular, authors discussed that aforementioned TAC-effects may be correlated with the AKT/NO signaling, given that TAC-induced AKT signaling activation is blunted in animals knock-out for AKAP1.

Several contributions point out the mechanisms of drugs that affect cardiovascular system targeting redox signaling pathways. The review by Varricchi et al. deals with cardiovascular toxicity (CTX) by chemotherapeutic agents which can alter redox homeostasis by increasing the production of ROS and reactive nitrogen species (RNS). The article reports that mitochondria are central targets for chemotherapeutic-induced CTX. The authors underline that, though, the last decade has witnessed intense research related to the molecular and biochemical mechanisms of CTX of antineoplastic drugs, experimental and clinical studies are urgently needed to balance safety and efficacy of novel cancer therapies. In this line, the paper by Riccio et al. demonstrates that the Na^+^ current inhibitor, *ranolazine*, is able to attenuate heart dysfunction induced by trastuzumab in animal and cellular models. The authors suggest that the cardioprotective role of ranolazine might be due to the blunting of ROS production induced by trastuzumab, as demonstrated *in vitro*. About drugs with cardioprotective effects, the work by Vieceli Dalla Sega et al. demonstrates that *ticagrelor* is able to lower circulating epidermal growth factor (EGF) which, in turn, leads to a better generation of NO in the vascular endothelium. The authors suggest that the capacity of ticagrelor in stabilizing platelets is also responsible for the lower release of EGF by platelets through a mechanism mediated by P2Y_12_. Taken together, data here presented indicate that–in addition to previously identified mechanisms like augmented adenosine bioavailability-the improvement of ticagrelor of endothelial function may depend on its greater efficacy in decreasing platelet reactivity. Interestingly, Russo et al. report a cardioprotective role for healthy platelets mediated by sphingosine-1-phosphate-dependent pathways, in the context of myocardial I/R. This cardioprotective effect is lost by platelets derived from poorly controlled diabetic patients and seems inversely correlated with the redox status and the reactivity of platelets. Antiplatelet agents might exploit the cardioprotective potentialities of platelets. Varga et al. in their original article suggest a role for NADPH oxidase (NOX) in ROS production during heart failure. They report that NOX4 undergoes extensive alternative splicing in human hearts, which gives rise to the expression of different enzyme isoforms. In particular, the full-length NOX4 is significantly upregulated in ischemic cardiomyopathy. These results may revive the development of NOX inhibitors based on the significant novel knowledge on the modulation of NOX activity, which may facilitate the targeting of NOXs in various diseases including myocardial infarction. Interestingly, Nydegger et al. in their elegant research work have shown that in the hypoxia-mediated model of pulmonary hypertension, modulation of the NO-cGMP pathway by *sildenafil* contrasts pulmonary vascular and right ventricle remodeling by an action that does not only encompass the canonical vasomodulatory effect but involves the modulation of several biochemical pathways. The potential role of phosphodiesterase-5 for long-term treatment, and perhaps prevention, of pulmonary hypertension is suggested and it is surely worthy of further investigation. The interesting original article by Rocca et al. demonstrates the cardioprotective role of the G protein-coupled estrogen receptor (GPER) expressed in the cardiovascular system, and of its selective ligand G1 through Notch signaling pathways in female hearts. The main finding of the study is the role of GPER in mediating the preconditioning mechanisms in normotensive and hypertensive conditions that protect the myocardium from I/R injury. G1-induced protection open new perspectives in the treatment of the myocardial ischemic injury. In their original article, Andreadou et al. show that *empagliflozin* (EMPA), a drug approved for type 2 diabetes management, reduces infarct size after I/R in mice and increase cell survival and ATP levels in rat embryonic-heart-derived cardiomyoblasts (H9C2) and endothelial cells (ECs). The protective effects of EMPA in mice are dependent on STAT3 activation and seem associated with reduced levels of malondialdehyde, myocardial iNOS, and interleukin-6 expression.

Recently, a great effort has been made to clarify the role of natural substances and/or antioxidants taken with diet or as food suppliers, in the prevention or treatment of cardiovascular diseases. On this topic, Sorriento et al. focus on antioxidants and in particular on *vitamin D* as anti-hypertensive agents. Arterial hypertension seems to depend on an imbalance between the production of ROS/RNS and the antioxidant defense mechanisms. The association between vitamin D deficiency and hypertension is strongly supported by literature suggesting that the supplementation of vitamin D could really become a therapeutic strategy for hypertension if an accurate selection of patients will be made. The authors propose that PTH levels, that regulate and are regulated by vitamin D, could be an important discriminating parameter in the selection of patients that could be sensitive to vitamin D supplementation. Thus, according to authors, vitamin D represents an antioxidant that is worthwhile to further investigate. Another compound that deserves to be studied is the *melatonin*. In their review article, Jiki et al. critically discuss the cardiovascular benefits of dietary melatonin. The authors report and discuss the papers on the effects of melatonin in different conditions, including hypertension and I/R injury. The issue at moment is: how can we increase the level of melatonin in human blood? Preclinical studies suggest that melatonin, given at dietary levels, confers cardioprotection. Circulating melatonin levels may have antioxidant capacity. However, there are many contradictory observations, still requiring responses. The original paper by Mastantuono et al. describes the effects of another natural antioxidant, the anthocyanin *cyanidin*. Studying rat pial microvascular changes due to cerebral blood flow reduction and recovery, the authors describe the protective mechanisms of this compound. Based on the results, they conclude that cyanidin protects cerebral microvasculature against vascular insult. Protection is elicited by recruiting the NO generation and a reducing ROS generation, thus preserving vascular permeability and vasodilation. Many pathological conditions, including hyperglycemia, may alter endothelial function through ROS/RNS overproduction. The paper by Querio et al. shows the antioxidant properties of *chamazulene*, a bioactive compound present in chamomile essential oil, in bovine aortic endothelial cells exposed to high glucose, and hydrogen peroxide-mediated oxidative stress. Their data suggest a possible use of this compound as a protective agent against endothelial injury.

The connection between cardiovascular system dysfunction and neurodegeneration is highlighted by Venturelli et al. that emphasize the importance of changes in NO bioavailability, cortical, extra-cranial, and peripheral blood flow in patients with Alzheimer's Disease (AD). The authors believe that these are phenomena primarily associated with AD and are not simply correlated with aging. Indeed, a relationship between AD and vascular impairment till to the more advanced phases of AD is described. Therefore, the link between cardiovascular and the central nervous system degenerative processes may be the depletion of endogenous NO. Since current AD treatments targeting Aß show very limited efficacy, potential new therapeutic approaches aimed to ameliorate the circulatory impairment and the depletion of NO bioavailability might be of pivotal interest for AD, and may reduce the high costs of patients' care. The study by Firinu et al. expands this concept by showing a significant decrease in endothelial function in another pathologic condition, namely the hereditary angioedema (HAE). In these patients, during the symptom-free period, a strong correlation between flow-mediated dilatation and *asymmetric dimethylarginine*, a strong inhibition of NO synthesis, was observed. This is in line with the described association of HAE and early atherosclerosis.

The topic includes also other two articles that deal with central nervous system diseases, emphasizing the cardiovascular aspects and the link with oxidative stress. In their mini-review, Paternò and Chillon discuss the similarity between two diseases of the CNS, ischemic stroke, and multiple sclerosis focusing especially on the astrocyte and neuroinflammation hallmarks shared by the two pathologies. Interestingly, the mini-review also highlight the astrocyte and neuroinflammation-targeted-strategies for the treatment of stroke and multiple sclerosis. The paper by Messina et al. suggests the need to broaden horizons and the study target on Autism spectrum disorders (ASD), including oxidative stress, neurotransmitters evaluation, and sympathetic activity measurements also related to cardiac functions. Sleep problems in ASD are a prominent feature, considering the role of orexins (A and B) in wake-sleep circadian rhythm, it is possible to speculate that ASD subjects may present a dysregulation in orexinergic neurotransmission. In this context may be explained the cerebral metabolism increasing and the autonomic hyperfunctioning in ASD sustained by high Orexin A levels.

Finally, in the special issue are included two reviews that may open new perspectives. In their interesting review article, Deidda et al. report several studies adopting a metabolomic approach that eventually could be helpful in elucidating mechanisms involved in redox and nitrosative reactions in relation to cardiovascular disease. These pieces of information may be of significant interest for both translational values and for improving an update of the protocols on metabolomics methods in cardiovascular diseases. By referring to teleost fish as paradigms of hypoxia- and anoxia-tolerance, Gattuso et al. illustrate cardiac strategies that, by involving nitric oxide and its metabolites, play a critical role in the adaptive responses to O_2_ limitation. Authors emphasize the power of the teleost heart as a bioassay to decipher the intricate molecular networks that crucially balance tissue O_2_ supply and demand. Information in this direction may be of significance also in a translational perspective for human cardioprotection and perhaps in hypoxia-mediated pulmonary hypertension.

The above-referenced articles are a clear demonstration that the research topic reached the aim of presenting the point of view of many scientists working in the field of redox biomedicine. The papers approached the topic from different points of view and at different levels, from basic to translational research. We hope these articles can contribute to the development of new ideas and advancements in the field of redox and nitrosative signaling in the control of normal cardiovascular functions and their disruption in diseases.

## Author contributions

All authors listed have made a substantial, direct and intellectual contribution to the work, and approved it for publication.

### Conflict of interest statement

The authors declare that the research was conducted in the absence of any commercial or financial relationships that could be construed as a potential conflict of interest.

